# Intronic Variant in *CNTNAP2* Gene in a Boy With Remarkable Conduct Disorder, Minor Facial Features, Mild Intellectual Disability, and Seizures

**DOI:** 10.3389/fped.2020.00550

**Published:** 2020-09-11

**Authors:** Raffaele Falsaperla, Xena Giada Pappalardo, Catia Romano, Simona Domenica Marino, Giovanni Corsello, Martino Ruggieri, Enrico Parano, Piero Pavone

**Affiliations:** ^1^Unit of Neonatology, University Hospital “Policlinico-Vittorio Emanuele, ” Catania, Italy; ^2^National Council of Research, Institute for Biomedical Research and Innovation (IRIB), Catania, Italy; ^3^Department of Biomedical and Biotechnological Sciences (BIOMETEC), University of Catania, Catania, Italy; ^4^Unit of Pediatrics and Pediatric Emergency, University Hospital “Policlinico-Vittorio Emanuele, ” Catania, Italy; ^5^Department of Sciences for Health Promotion and Mother and Child Care “G. D'Alessandro, ” University of Palermo, Palermo, Italy

**Keywords:** *CNTNAP2* gene, intronic copy number variant, conduct disorder (CD), epilepsy, intellectual disability (ID)

## Abstract

**Introduction:** Mutations in the contactin-associated protein-like 2 (*CNTNAP2*) gene (MIM#604569) encoding for CASPR2, a cell adhesion protein of the neurexin family, are known to be associated with autism, intellectual disability, and other neuropsychiatric disorders. A set of intronic deletions of *CNTNAP2* gene has also been suggested to have a causative role in individuals with a wide phenotypic spectrum, including Pitt-Hopkins syndrome, cortical dysplasia–focal epilepsy syndrome, Tourette syndrome, language dysfunction, and abnormal behavioral manifestations.

**Case presentation:** A 10-years-old boy was referred to the hospital with mild intellectual disability and language impairment. Moreover, the child exhibited minor facial features, epileptic seizures, and notable behavioral abnormalities including impulsivity, aggressivity, and hyperactivity suggestive of the diagnosis of disruptive, impulse-control and conduct disorder (CD). Array comparative genomic hybridization (CGH) revealed a copy number variant (CNV) deletion in the first intron of *CNTNAP2* gene inherited from a healthy father.

**Conclusions:** A comprehensive description of the phenotypic features of the child is provided, revealing a distinct and remarkable alteration of social behavior not previously reported in individuals affected by disorders related to *CNTNAP2* gene disruptions. A possible causative link between the deletion of a non-coding regulatory region and the symptoms presented by the boy has been advanced.

## Introduction

A new strong candidate gene for psychiatric and language disorders is contactin-associated protein-like 2 (*CNTNAP2*) gene (MIM#604569). *CNTNAP2* is a member of the neurexin family and consists of a transcript of 24 exons encoding for CASPR2 protein, which functions as a cell-adhesion molecule in many neuronal activities, such as neuronal migration, dendritic arborization, and synaptic transmission. The main role of CASPR2 is the conduction of axon potentials and the clustering of voltage-gated potassium channels at the juxtaparanodes in both myelinated axons of the spinal cord and of the central nervous system (1, 2). However, the high expression of the protein in Broca's area and other perisylvian regions is consistent with the emerging role in normal language development and social communication (3, 4). By its size, spanning 2.3 Mb at chromosomal region 7q35-36, *CNTNAP2* is a target for a wide variety of mutations and structural rearrangements, including copy number variants (CNVs). Some intronic CNV deletions of *CNTNAP2* gene have been reported in individuals with Pitt-Hopkins syndrome (PTHS) and cortical dysplasia–focal epilepsy syndrome (CDFES), epilepsy with auditory features (EAF), autism spectrum disorder (ASD) and intellectual disability (ID), speech impairment, Tourette syndrome (TS), and abnormal behavioral manifestations (5–9). Interestingly, variants found in the first intronic region of *CNTNAP2* have been shown to lead to the loss of critical regulatory elements of some conserved transcription factor (TF) binding sites (TFBSs) involved in language and social-emotional development.

Here, we report a child presenting with neuropsychiatric disturbances including notable behavioral disorders consisting of frequent and severe outbursts of impulsivity, aggressivity, and hyperactivity suggestive of the diagnosis of “disruptive, impulse-control, and conduct disorder (CD)” and TS. Moreover, the child exhibited minor facial features, mild ID, language impairment, and epileptic seizures. Molecular investigation carried out by array comparative genomic hybridization (aCGH) revealed a CNV deletion spanning 95.4 kb in intron 1 of *CNTNAP2* gene inherited from a healthy father. A possible causative link between the deletion of a non-coding regulatory region and the clinical manifestations observed in the boy has been raised.

## Materials and Methods

### Case Presentation

A 10-years-old boy is the second child of healthy unrelated Italian parents. Both older half-siblings, an 18-years-old boy born from the father's previous marriage and a 16-years-old sister are healthy. In the paternal line, there is a history of family members affected by polyposis intestinalis with early deaths.

At gestation, the mother was 34 years old and the father was 40 years old. The mother denied having had complications during the gestation and reported normal fetal movements. Intrauterine ultrasound did not show fetal anomalies. The child was born at 40 weeks of gestation by cesarean section as in the previous delivery. Birth weight was 3.6 kg, length 51 cm, and head circumference 35 cm (all within normal limits). The Apgar scores were eight at one and 10 at 5 min. At birth, neither clinical signs nor notable facial features were noticed, and the child was discharged by the hospital in good condition.

**Figure 1:**
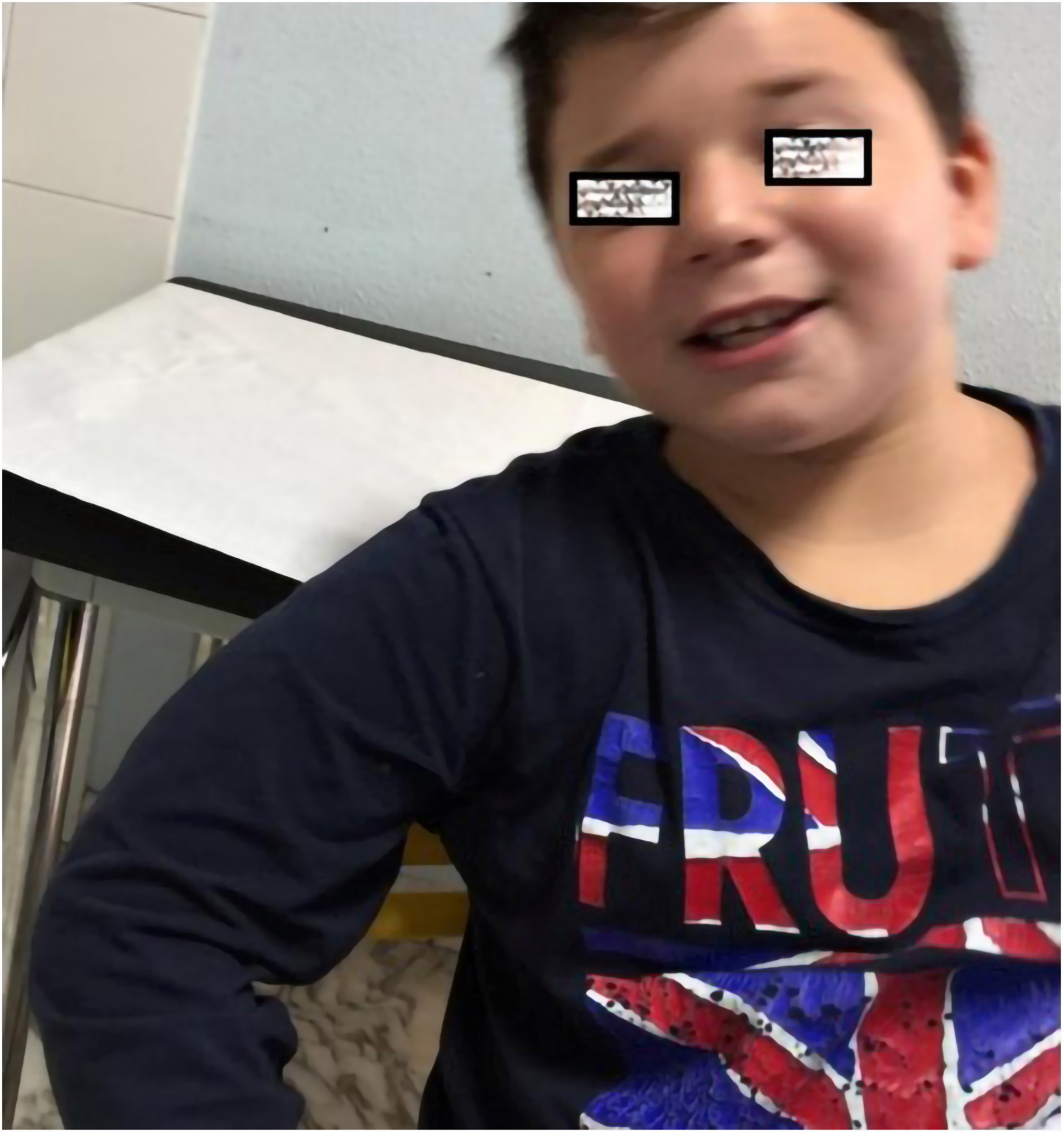
Photo showing a 10-years-old boy with sparse eyebrows, short nose, long flat philtrum and thin lips.

**Figure 2:**
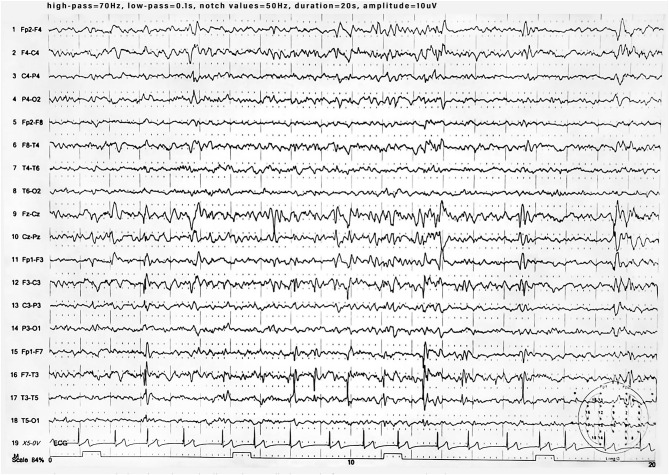
EEG during sleep showing spike and wave discharges in fronto-centro-temporal region.

During the 1st months, he exhibited normal motor development but had speech delay, which started at the age of 28 months with the pronunciation of a single word. At the age of 3 years, the parents noted that the child was extremely overactive with mood swings and difficulty in sleeping. The anomalous behavior became progressively more evident, and at the age of 4 years, he was remarkably aggressive. At the age of 5 years, he was hospitalized in North Italy due to developmental delay, frequent insomnia episodes, stereotypic movements (lateral swinging), and phonologic disturbances. During hospitalization in that center, routine examination including thyroid markers, ECG, and brain MRI were performed with normal results. In the Leiter-R test (non-verbal cognitive capacity), brief IQ of 85 was registered, and the “Child Behavioral Check List” (CBCL) showed affective impairment, anxiety, hyperactivity, and obsessive behavior. The child was discharged with the diagnosis of behavioral disturbance with hyperactivity, phonologic disturbances, and EEG alterations. At the age of 8 and 9 years, right-sided focal tonic seizures lasting a few minutes were recorded. Treatment with valproate at 20 mg/kg/day was started with good drug response. During the subsequent years, he was followed up as an outpatient at the Pediatric Unit and Pediatric Emergency Unit, University Hospital “Policlinico-Vittorio Emanuele,” Catania, Italy, since he showed mild speech impairment and episodes of aggressivity against parents, teachers, and peers. In one of these episodes, he caused injury to one of his teachers. Risperidone 1 mg/day was started but irregularly administered with poor results. At the age of 10 years, he was admitted to this institution for a clinical workup. His weight was 51 kg (>90th percentile), length 143 cm (75th percentile), and head circumference 55 cm (90th percentile). On physical examination, minor feature anomalies were noticed and consisted of upslanting palpebral fissures, sparse eyebrows, short nose, flat philtrum, thin lips, and wide earlobes (Figure 1). Hands and feet were short. The heart, thorax, abdomen, and internal organs were normal. Neurological examination was normal, and patellar tendon reflexes were normally elicited. EEG during wakefulness and during sleep showed spike and wave discharges in the fronto-centro-temporal region, and during eye opening, photic stimulation and hyperventilation was unchanged (Figure 2). A fundus examination and hearing exploration were normal. At the age of 10 years, brief IQ was 85. No more seizures were reported, and treatment with valproate was withdrawn. Due to the frequent chronic episodes of diarrhea and familiar history of polyposis intestinalis, an abdominal ultrasound exam and esophagogastroduodenoscopy (EDG) were performed, and tissue samples were examined. The EDG did not show anomalies, and the macroscopic sample analysis of the duodenal tissue was normal. The neuropsychiatric evaluation of the child displayed several dysfunctions. His cognitive and behavioral profile consisted of a mild ID and high score of generalized anxiety and depression with intrusive thoughts. Complex, chronic motor tics were seen in the child in association with signs of obsessive–compulsive disorder (TS). Self-esteem and social relation result compromised. Verbal and physical aggressivity and impulsivity were particularly expressed. The final psychiatric diagnosis was disruptive, impulse-control and CD. The results of the neuropsychiatric evaluation of proband and parents are reported in Table 1(A–J). At last examination, at 11 years old, seizures were not reported, while behavioral impairments were unmodified.

**Table 1:** (A–J). Neuropsychological assessment of proband (A–G) and both parents (H–J).

**A. WISC-I** **(Wechsler Intelligence Scale for Children)**		**B. CY-BOSH (Children's Yale–Brown Obsessive–Compulsive Scale)**		
Verbal comprehension	70	Subtotal Obsessions	8	
Perceptual reasoning	71	Subtotal Compulsions	16	
Working memory	64	Total	24 (low score)	
Processing speed	88	**C. General Score anxiety disorders**		
Standard score range for WISC-IV: 64		Total 19	points T 66 (low score)	
**D. MASC (Multidimensional Anxiety Scale for Children) test**				
Physical symptoms	Total 19	Points T 63 (borderline)		
Harm avoidance	Total 20	Points T 57 (normal)		
Social anxiety	Total 18	Points T 65 (low score)		
Separation/Panic	Total 20	Points T 82 (low score)		
Total	Total 77	Points T 72 (positive)		
**E. Children's Depression Inventory (CDI)**				
Cutoff	15			
Total	24 (low score)			
**F. Small bell Test (measure selective and sustained attention)**				
Speed	Total 25 (between 50 and 75th percentile)			
Accuracy	Total 57 (25th percentile)			
**G. Paper and Pencil test**				
Insecure child with decreased auto-esteem and social relation				
**H. CBCL (Child Behavior Checklist)**				
		**Interview to the father**	**Interview to the mother**	
Total Score		pT 73 low score	pT 63 (borderline)	
Internalizing problems		pT 71 low score	pT 64	
Externalizing problems		pT 74 low score	pT 66	
Thought problem Scale		pT 79	pT 67	
Aggressive behavior Scale		pT 76	pT 63	
Anxiety-Depression		pT 74	pT 66	
**I. SNAP-IV (Swanson, Nolan and Pelham-IV)**				
	**Interview to the father**	**Cutoff**	**Interview to the mother**	**Cutoff**
Inattention	Total 15 –> 1,66	1,78	Total 12 –> 1,33	1,78
Hyperactivity/Impulsivity	Total 17 –> 1,88	1,44	Total 14 –> 1,55	1.44
Oppositional defiant	Total 15 –> 1,87	1,88	Total 10 –> 1,25	1,88
**J. SCOD IV (Rating scale for disruptive behavioral subscale)**				
	**Interview to the father**		**Interview to the mother**	
Inattention	Percentile 95–100		Percentile 95–100	
Defiant disorder	Percentile 95–100		Percentile 95–100	
Conduct disorder	Percentile 95–100		Percentile 95–100	

### Microarray Experiment and Data Analysis

Genomic DNA was isolated from peripheral blood of the proband, together with the proband's mother and father. aCGH was performed by CytoSure ISCA 8 × 60 k array from Oxford Gene Technology (OGT) according to the manufacturer's recommendations (Agilent Technologies, Santa Clara, CA). aCGH data were analyzed and interpreted using Cytosure software (GRCh38 assembly) provided by OGT.

Aiming at exploring and better interpreting the phenotype linked with the proband's CNV, we used the publicly available patient data on the Database of Genomic Variants (DGV) (dgv.tcag.ca), DECIPHER web-based resource (decipher.sanger.ac.uk), CNV dataset from Clinical Genome Resource (ClinGen), and Morbidity Map of Developmental Delay displayed at UCSC Genome Browser (genome.ucsc.edu).

The molecular karyotype revealed that the proband carries a CNV deletion of 95.4 kb in 7q35(146,271,924-146,367,324)x1 inherited from the healthy father. The microdeletion detected in the first intronic region of the *CNTNAP2* gene has been previously reported in the DGV database.

## Discussion

The young boy presented with minor facial dysmorphism, mild ID, speech impairment, sleep disorders, and severe behavioral disturbances and TS. Two episodes of partial epileptic seizures were also recorded. Behavioral disturbances were impressive, and the child exhibited frequent and severe outbursts of aggressivity, impulsivity, and hyperactivity, which led to the diagnosis of disruptive, impulse-control and CD. Much of the clinical signs presented in the child have been also reported in patients affected by CASPR2-deficiency disorder (CDD) associated with language impairment, notable impaired behavior and CD, TS, epileptic seizures, moderate ID, and poor social interactions (8, 10, 11). aCGH analysis revealed a small CNV deletion in intron 1 of *CNTNAP2* gene inherited from the unaffected father. The incomplete penetrance of the CNV variant may not exclude the pathogenic link between the microdeletion and disruption of gene-regulatory and protein interactions underlying some neural and behavioral pathways involved in learning ability and language and social behaviors (11–13). Deletions with similar size (e.g., essv12997739) of the variant in question have been reported in population, but their pathogenicity and phenotypic contribution are still uncertain. A wide variety of intronic variants of *CNTNAP2* gene has been reported in patients with severe ID, autistic behavior, epilepsy, and breathing anomalies that phenotypically overlap with PTHS (14, 15). Additional reports have also been found in individuals with epilepsy (16), EAF (7), epilepsy, and schizophrenia in three non-related Caucasian patients (17). Moreover, Strauss et al. (18) reported children with cortical dysplasia, focal epilepsy, relative macrocephaly, and diminished deep-tendon reflexes in association with language regression, hyperactivity, and impulsive and aggressive behavior. Speech disorders, behavioral disturbances, and other neuropsychiatric disorders have been identified in patients with alterations in the *CNTNAP2* gene (12, 19–22). Verkerk et al. (23) described a family with members affected by TS and obsessive–compulsive disorder (OCD), in which mutations in *CNTNAP2* gene expression have been found to alter the distribution of the K(+) channels in the nervous system with abnormal conduction and/or repolarization of active potential, leading to cause anomalous motor movements observed in individuals affected by TS. In contrast with this finding, no individuals with clinical evidence of TS have been reported by Belloso et al. (24) in a family with a balanced reciprocal translocation (t7;15)(q35:q26.1). The first intron of *CNTNAP2* is known to be a susceptible locus for structural rearrangements that may influence the genetic modulation of the gene since it contains some conserved regulatory regions implicated in the transcriptional network of neurodevelopmental processes regulating language skills and social-emotional functioning. Some studies have experimentally identified consensus binding sequences for a selected set of TFs, such as *STOX1A* (8), *TCF4* (25), *FOXP1*, and *FOXP2* (26, 27), which can influence the transcriptional control of the gene and interact with *CNTNAP2* to co-regulate the developmental pathways of human speech and social behavior.

### Limitations

Our study has some limitations. First is that the described CNV may be a combined effect of additional rare variants, which are not excluded in the present case, and should be investigated by whole-exome sequencing (WES). Secondly, with regard to the quantitative PCR validation, *CNTNAP2* gene dosage variation was not included. However, we neglected to correlate the effect of CNV loss with the gene expression level to examine alterations in mRNA synthesis and the activity of Caspr2 protein. The impact of the intronic deletion in the transcriptional regulatory network of CNTNAP2 that correlated with the analysis of FOXP interactome warrants additional investigations. Our study might serve as a first step to identify a non-coding regulatory variant enriched for potentially important TFs implicated in neurodevelopment. We believe that it would be important to determine whether (i) the effect of CNV loss may affect variations in the *CNTNAP2*-encoded CASPR2 protein (e.g., splice site mutations, posttranslational modifications) and whether (ii) the clinical phenotype may depend on the association between CNV loss and DNA methylation pattern of gene promoter and intron 1.

## Concluding Remarks

The present study deems that regardless of sufficient evidence to conclude that the intronic deletion of *CNTNAP2* has a potential contribution in the diagnosis, there are some findings for believing in a likely positive correlation between the clinical presentation of the child and individuals affected by CDD.

## Data Availability Statement

The data used to support the findings of this study may be released upon application to the corresponding author who can be contacted at ppavone@unict.it.

## Ethics Statement

The study was conducted ethically in accordance with the World Medical Association Declaration of Helsinki and was approved by the ethic committee of the University of Catania, Italy (Ethical Committee Catania 1 Clinical Registration n. 95/2018/PO). Informed consent was obtained from parents of the proband.

## Author Contributions

RF and PP worked with and helped gather patient data and drafted and redrafted the present manuscript. XP helped analyze the genetic data and interpret the literature relevant to the mutation. CR performed the neuropsychological assessment. CR and SM contributed to the clinical understanding of the case and revised the manuscript. GC, MR, and EP were called as consultants regarding the clinical diagnosis and reviewed the manuscript. All authors read and approved the final manuscript.

## Conflict of Interest

The authors declare that the research was conducted in the absence of any commercial or financial relationships that could be construed as a potential conflict of interest.

## Abbreviations

aCGH: array comparative genome hybridization
ASD: autism spectrum disorder
CCD: CASPR2-deficiency disorder
CD: conduct disorder
*CNTNAP2*: contactin-associated protein-like 2
CNV: copy number variant
CDFES: cortical dysplasia–focal epilepsy syndrome
EAFs: epilepsy and auditory features
EDG: esophagogastroduodenoscopy
*ESR1*: estrogen receptor alpha gene
*FOXP1*: forkhead box P1
*FOXP2*: forkhead box P2
ID: intellectual disability
OCD: obsessive–compulsive disorder
PTHS: Pitt-Hopkins syndrome
*STOX1A*: storkhead box 1A
TFBS: transcription factor binding site
TF: transcription factor
TCF4: transcription factor 4
TS: Tourette syndrome.
